# Contribution of Manure-Spreading Operations to Bioaerosols and Antibiotic Resistance Genes’ Emission

**DOI:** 10.3390/microorganisms11071797

**Published:** 2023-07-13

**Authors:** Mahsa Baghdadi, Patrick Brassard, Stéphane Godbout, Valérie Létourneau, Nathalie Turgeon, Florent Rossi, Émie Lachance, Marc Veillette, Marie-Lou Gaucher, Caroline Duchaine

**Affiliations:** 1 Département de Biochimie, de Microbiologie et de Bio-Informatique, Faculté des Sciences et de Génie, Université Laval, Québec, QC G1V 0A6, Canada; mahsa.baghdadi.1@ulaval.ca (M.B.); 2 Centre de Recherche de L’institut de Cardiologie et de Pneumologie de Québec, Québec, QC G1V 4G5, Canada; 3 Institut de Recherche et de Développement en Agroenvironnement, Québec, QC G1P 3W8, Canada; 4 Chaire de Recherche en Salubrité des Viandes, Département de Pathologie et Microbiologie, Faculté de Médecine Vétérinaire, Université de Montréal, Saint-Hyacinthe, QC J2S 2M2, Canada; 5 Canada Research Chair on Bioaerosols, Québec, QC G1V 4G5, Canada

**Keywords:** bioaerosols, antimicrobial resistance genes, manure spreading

## Abstract

Manure spreading from farm animals can release antibiotic-resistant bacteria (ARB) carrying antimicrobial resistance genes (ARGs) into the air, posing a potential threat to human and animal health due to the intensive use of antibiotics in the livestock industry. This study analyzed the effect of different manure types and spreading methods on airborne bacterial emissions and antibiotic resistance genes in a controlled setting. Cow, poultry manure, and pig slurry were spread in a confined environment using two types of spreaders (splash plate and dribble bar), and the resulting emissions were collected before, during, and after spreading using high-volume air samplers coupled to a particle counter. Total bacteria, fecal indicators, and a total of 38 different subtypes of ARGs were further quantified by qPCR. Spreading poultry manure resulted in the highest emission rates of total bacteria (10^11^ 16S gene copies/kg manure spread), *Archaea* (10^6^ 16S gene copies/kg manure), *Enterococcus* (10^5^ 16S gene copies/kg manure), and *E. coli* (10^4^ 16S gene copies/kg manure), followed by cow manure and pig slurry with splash plates and the dribble bar. Manure spreading was associated with the highest rates of airborne aminoglycoside genes for cow and poultry (10^6^ gene copies/kg manure), followed by pig slurry (10^4^ gene copies/kg manure). This study shows that the type of manure and spreading equipment can affect the emission rates of airborne bacteria, and ARGs.

## 1. Introduction

The problem of antibiotic resistance has become a major concern in modern societies [1,2] due to antimicrobial-resistant bacteria (ARB), which are found everywhere in the environment, including water, soil, and air [3]. While a fraction of them is naturally occurring as the result of millions of years of coevolution dynamics, human activity has greatly contributed to their increases. Agriculture in particular, could be an important vector of antimicrobial resistance genes (ARGs) into the surrounding environments [4]. The gastrointestinal system of livestock contains high levels of microorganismsfound either temporarily or continuously [5], comprising viruses (e.g., animal viruses, bacterial viruses, or phages) and bacteria (e.g., *E. coli, Enterococcus*)*,* as well as *Archaea*, that can act as a potential source of zoonotic pathogens and antimicrobial resistance genes to be transmitted to the environment through untreated manure [5]. As a result, a wide range of concentrations of ARB and related ARGs are commonly reported in soils fertilized with manure [6].

During and after the spreading of manure, a fraction of the bacteria, viruses, fungi, and protozoa [5,7], including their related ARGs, can possibly aerosolize and disperse in a wider range [8]. Based on our current experiment, the application of 1.5 m^3^ of manure would lead to the emission of ~10^9^ copies of ARGs/kg of manure into the air, making it an important source of biological pollution on a global scale, with possible adverse effects toward human health [8], especially for livestock workers. Airborne concentrations of bacteria, fungi, endotoxins, and antibiotic-resistant bacteria have also been widely reported. For instance, concentrations of airborne *Staphylococcus* spp. were significantly higher downwind rather than upwind of the swine and poultry farms (up to 1.9 × 10^3^ cfu/m^3^) [9,10,11].

All kinds of farming operations can lead to microorganisms’ aerosolization [5,7,12]. Manure spreading, in particular, can be expected to be the major contributor to these emissions, which are then expected to decrease once spreading is complete. Therefore, the use of the appropriate spreader can be critical. So far, the equipment used for spreading manure mostly depends on the type of manure, whether it is liquid or solid. Solid manure is often spread using box spreaders pulled by tractors or trucks, with side discharge spreaders and box spreaders with vertical, horizontal, and spinner beaters being the most used [13,14]. For liquid manure, a tank wagon with splash plates has traditionally been used. Every method obviously has its pros and cons, depending on the context and the type of manure, but research remains severely limited. For example, according to the FarmTech website [15], using drop hoses for liquid manure can lead to a more uniform spreading, but with no indication about bioaerosol emissions. Indeed, these can be difficult to assess in that context, especially outdoors, where environmental conditions such as the wind direction and speed can drastically affect the observed emissions.

The current study aims to assess the effect of different types of manure (solid versus liquid manure, poultry versus cow manure) and spreaders (horizontal beater for solid cow and poultry manure, splash plate and dribble bar for pig slurry) on bioaerosol emissions. The experiment was conducted in a wind tunnel, providing a partially controlled environment, therefore allowing for a more accurate estimation of the emissions and robust comparisons between different manure types (solid and liquid manure) and spreaders [16,17,18]. The airborne concentrations of bacteria, and ARGs, as well as fecal indicators such as *E. coli*, *Enterococcus*, *Archaea*, and the phage *vB_AviM_AVP* of *A. viridans,* that are known proxies of fecal continuation, were systematically measured during the spreading and immediately after, and further related to their expected sources within the manure. Overall, the present study will provide significant insights on factors that can influence bioaerosol emissions related to the spreading of manure, that could be helpful for authorities in managing the risk assessment toward livestock workers.

## 2. Materials and Methods

### 2.1. Experimental Design and Manure Spreading

A greenhouse (8 m width × 30 m length × 4 m height) located at the Research and Development Institute for the Agri-Environment (IRDA) experimental farm (St-Lambert-de-Lauzon, QC, Canada) was adapted as a field-scale wind tunnel to spread manure in a controlled environment [19]. The wind tunnel is equipped with 10 fans (24-inch diameter each) blowing air from the inside to the outside at an airflow of 8 m^3^ per second, equivalent to an air change rate of 41 times per hour (Figure S1 and Section S1). Three types of manure were spread over a small-scale loamy soil plot (8 m width × 22 m length × 0.6 m height): solid beef cow manure, solid poultry manure, and liquid pig slurry. Poultry manure (without litter) was collected at an egg production facility, where it was pre-dried on a belt conveyor at ambient temperature. Beef cow manure was stored in a pile at the farm for 3 to 6 weeks (a composting reaction was started) before being spread. Finally, pig slurry was collected from the pit of a swine finishing building (Table S1). It is also noteworthy that no antibiotic is used on a regular basis on the farms providing manure for our experiments. However, antibiotic treatments may have been used for treating animals in the farms.

The three types of manure were separately applied during distinct “campaigns” to avoid cross-contaminations. Each of the aforementioned “campaigns” consisted of six consecutive spreads of one type of manure over six different days. Each spreading was started the same day as the air samplings (the “during spreading”), and the corresponding manure in the greenhouse was systematically removed after the last air sampling (the “after spreading”) by scrapping the first centimeters of dirt by shoveling the surface of the soil and removing the manure.

Different spreaders were used according to the type of manure (solid versus liquid). Specifically, solid beef cow and poultry manures were spread out at rate of 1.8 and 0.51 kg m^−2^, respectively, using a horizontal beater spreader (MX50G model, Wallenstein Equipment Inc., Wallenstein, ON, Canada). Liquid pig slurry was spread out at a rate of 3.15 kg m^−2^ using a splash plate device and a low spreading toolbar equipped with dribble bars (Figure S2). Each spreading (cow manure, poultry manure, pig slurry with splash plate, and pig slurry with dribble bars) was repeated six times regardless of the external weather conditions (temperature, relative humidity), but in the absence of any precipitation.

### 2.2. Sampling of Manure and Air

Samples of manure were collected prior to each spreading to further characterize their physical physicochemical properties and their content in fecal indicators and ARGs. Three samples of 50 g of cow or poultry manure were taken from different zones and layers of the pile with sterile scoops and put into sterile bags, whereas 50 mL of pig slurry was directly collected into 50 mL tubes. Samples were then kept at 4 °C until further laboratory analyses.

For each manure- and slurry-spreading trial, air samples were collected in the downwind and at the exhaust of the wind tunnel for three periods of 20 min, starting: (1) 20 min before spreading, herein called “before spreading”, (2) at the start of spreading, herein called “during spreading”, and (3) 20 min after the spreading started, herein called “after spreading”. Air was sampled using a SASS^®^3100 Dry Air Sampler (300 L/min, Research International Inc., Monroe, WA, USA) and a SASS^®^4100 Two-Stage Aerosol Collector (4000 L/min, Research International). Both air samplers allow collecting bioaerosols on an electrostatic filter. Field blanks—unused filters brought back from the field—were systematically performed onsite by placing a filter on an air sampler and removing it after 2 min. All air samples were kept at 4 °C until further analysis.

In addition, a particle counter (3 L/min, DustTrak™ DRX Aerosol Monitor, model 8534, TSI Incorporated, Shoreview, MN, USA) was used at the exhaust of the wind tunnel (downwind) and close to the bioaerosol samplers to measure the real-time emissions of PM1, PM2.5, PM4, PM10, and total airborne dust particles. Temperature, relative humidity, wind direction, and wind speed were monitored, and data were reported by Desbiens et al. [19]. Finally, during the spreading, measures of gas (CO_2_, CH_4_, N_2_O, NH_3_, H_2_S) and odor were also conducted, as described in Desbiens et al. [19].

### 2.3. Processing of Manure and Air Samples

Processing of manure samples differed according to the type of manure. Briefly, 25 g of solid manure (cow or poultry manure) was first homogenized in 200 mL of phosphate-buffered salt (PBS) 1× solution (supplemented with 0.05% Tween 20) using a paddle blender (1 min, 230 bpm, model 11-452-120, Fisher Scientific, Pittsburgh, PA, USA). As per liquid manure (pig slurry), samples were not homogenized in PBS buffer. Therefore, Aliquots of 500 µL of homogenates (cow and poultry manure) or 500 µL of pig slurry were then centrifuged once (20,817× *g*, 10 min). Pellets were kept at −20 °C until DNA extraction. Total DNA from air and manure samples was extracted using the DNeasy^®^ PowerLyser^®^ PowerSoil^®^ DNA extraction kit (QIAGEN, Mississauga, ON, Canada) according to the protocol established by the manufacturer. The purified DNA was eluted at 100 µL and stored at −20 °C until subsequent analyses.

Regarding air samples, particles on electrostatic SASS^®^ filters were first extracted using the SASS^®^3010 Particle Extractor (Research International, Inc.), along with 7 mL of sterile phosphate buffer (138 mM sodium chloride, 2.7 mM potassium chloride, 10.0 mM sodium phosphate, 15.4 mM sodium azide, and 0.8 mM Triton X-100). The resulting volume of the extracted samples (~6.5 mL) was then pelleted by successive centrifugations (20,817× *g*, 10 min) into 1.7 mL tubes. Pellets were stored at −20 °C until DNA extraction.

### 2.4. Quantification of Total Bacteria and Fecal Indicators Using Classic CFX qPCR

Airborne total bacteria, *E. coli, Enterococcus, Archaea,* and a phage of *Aerococcus* (phage *vB_AviM_AVP* of *Aerococcus viridans*) were quantified by PCR from DNA extracted from manure and SASS^®^3100 samples. Quantification was achieved by using a serial dilution of a plasmid containing the PCR-targeted sequence (10^6^ to 10^0^ copies/µL). More details about qPCR thermo-protocols (Table S2), primers, and probes are provided in the Supplementary Material (Tables S3 and S4). All qPCR runs were conducted on the CFX384 or the CFX96 Touch^TM^ real-time PCR Systems (Bio-Rad laboratories Mississauga, ON, Canada). Negative controls were included in each PCR assay, and all samples, including negative and positive controls, were tested in triplicate for total bacteria and in duplicates for fecal indicators and *Aerococcus* phage. Concentrations of bacteria, *Archaea,* and fecal indicators were then expressed in copies per cubic meter of air (copies/m^3^).

### 2.5. Quantification of ARGs Using the TAKARA Platform

Thirty-eight different subtypes of ARGs, providing resistance to eight different classes of antibiotics (beta-lactams, aminoglycosides, glycopeptides, quinolones, sulfonamides, tetracyclines, polymyxin, and macrolides) and three mobile genetic elements (MGEs) were quantified by PCR on a high-throughput qPCR Smartchip platform (Takara bio, San Jose, CA, USA) from DNA extracted from manure and SASS^®^4100 samples. qPCR primers for ARGs and MGEs were previously described by Nijhuis et al. and Stedfeld et al. [20,21]. PCR mixtures (200 nL per well) consisted of 1 × TB Green Gene Expression Master Mix platform (Takara bio, San Jose, CA, USA), 200 nM of each primer, and 100 nL of DNA template. The initial enzyme activation was performed at 95 °C for 10 min, and then 45 cycles of the following program were used for amplification: denaturation at 95 °C for 10 s and annealing at 60 °C for 30 s. A melting curve from 60 °C to 94 °C was generated. Raw qPCR results were analyzed using SmartChip qPCR Software Version 2.8.68. Wells with multiple melting peaks or with amplification efficiencies beyond the range (1.8–2.2) were discarded. A threshold cycle (CT) of 33 was used as the detection limit, and ARGs with amplification in a minimum of 2/3 replicates were regarded as positive. More details about the primers and probes are listed in Tables S3 and S4 [21,22].

### 2.6. Calculation of Emission Rates for Total Bacteria, Fecal Indicators, Aerococcus Phage, and ARGs

To obtain the emission rates at the time of the manure spreading or after (residual emission), concentrations of bacteria, *Archaea*, *E. coli*, *Enterococcus*, *Aerococcus* phage, and ARGs (copies/m^3^) of the “before spreading” air samples were first subtracted from concentrations of the “during spreading” air samples or from concentrations of the “after spreading” air samples, respectively. Emission rates (R) of the studied microorganism or ARG (in copies of genes/kg of manure) were then calculated using the following equation:

R = (C × Q × t)/(A × d)

where C is the airborne concentration increase from “before spreading” (copies/m^3^ of air), Q is the airflow rate of the wind tunnel (m^3^/min), t is the sampling time (min), A is the surface area of the soil on which manure was spread (m^2^), and d is the dose of manure applied (kg/m^2^).

### 2.7. Statistical Analyses

Airborne emission rate data were analyzed using a linear mixed model using two fixed factors. The three different types of manure with different spreading methods were associated with one of these factors, while the other factor was related to the time of spreading (during vs. after), and they were analyzed as repeated measures. An interaction term between these two fixed factors was added to the statistical model. The samplers were defined as a random factor nested in manure types. The dependence among residuals of repeated measurements from the same experimental unit was estimated with an unstructured variance–covariance association. Variables were log-transformed to fulfill the normality and variance assumptions. Since the data were correlated, the normality assumption was verified with the Shapiro–Wilk test using residuals from the statistical model and transformed by the Cholesky’s metric. The graphical representation of the marginal linear predictor with studentized residuals suggested the homogeneity of variances. The absolute number of total ARGs for different manure types during and after spreading was analyzed using the same statistical model proposed for airborne emission rate data. Antibiotic resistance genes’ (ARGs) emission rate data were analyzed using a non-parametric model, since many variables with non-detectable values (left censored) were reported and the normality and variance assumptions were never fulfilled after the investigation of many transformations. A non-parametric mixed statistical model on longitudinal data proposed by Brunner [23] was performed. The values were transformed by their ranks and the statistical model proposed previously was applied with corrections for *p*-values on the fixed factor. Emission rates of airborne dust particles were analyzed using a linear mixed model using two fixed factors. The four different types of manure were associated with one of these factors, while the other factor was associated with the time of spreading (before, during, and after), and they were analyzed as repeated measures. An interaction term between these two fixed factors was added to the statistical model. The samplers were defined as a random factor nested in manure types. The dependence among residuals of repeated measurements from the same experimental unit was estimated with an autoregressive covariance matrix structure. Since the data were correlated, the normality assumption was verified with the Shapiro–Wilk test using residuals from the statistical model and transformed by the Cholesky’s metric. The graphical representation of the marginal linear predictor with studentized residuals suggested the homogeneity of variances. The results were considered significant with *p*-values ≤ 0.05. The data were analyzed using the statistical package program SAS v9.4 (SAS Institute Inc., Cary, NC, USA).

## 3. Results

### 3.1. Bacteria, Archaea, Fecal Indicators, and ARGs in Manure

Manure is known as the main source of emitted bioaerosols. In the present study, the average concentration of total bacteria was found to be the highest in cow manure and pig slurry (7.61 × 10^13^, 9.29 × 10^13^, and 1.91 × 10^13^ 16S rRNA gene copies/g of dry matter, respectively) and the lowest in poultry manure (Table 1A). A similar observation can be made for *Archaea*, which was 2 log lower in the latter. Conversely, *Enterococcus*, *E. coli*, and the *Aerococcus* phage were the lowest in cow manure (1.91 × 10^7^, 7.65 × 10^4^, and 1.45 × 10^4^ 16S rRNA gene copies/g of dry matter, respectively), whereas the other types of manure displayed very similar concentrations (Table 1A).

**Figure 1:**
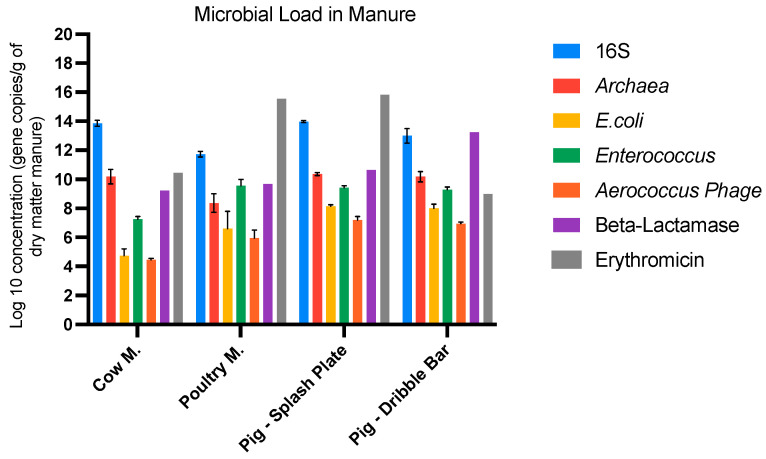
Concentration of total bacteria, fecal indicators, phage, and the most abundant ARG in the manure.

Beta-lactamase and tetracyclines, as well as sulfonamides, resistance genes were the most abundant ARGs in pig slurry with the dribble bar (1.70 × 10^13^, 1.42 × 10^12^, and 2.75 × 10^11^ gene copies/g of dry matter, respectively) (Table 1B). Erythromycin and tetracycline resistance genes were the most reported ARGs in pig slurry with the splash plate (6.72 × 10^15^, 2.31 × 10^12^, gene copies/g of dry matter, respectively). Erythromycin resistance genes had the highest concentration in poultry manure (3.52 × 10^15^ gene copies/g of dry matter). According to these results, on average, erythromycin and beta-lactamase resistance genes were the most abundant in manure for most of the experiments (10^15^ and 10^12^ gene copies/g of dry matter) (Figure 1).

### 3.2. Airborne Emissions of Total Bacteria and Dust Particles

**Figure 2:**
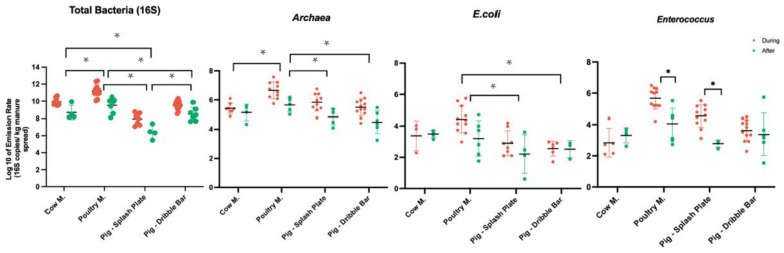
Emission rates of total bacteria and fecal indicators (16S rRNA gene copies/kg). Bracket shows a significant difference between the types of manure, whereas the asterisk (*) shows a statistical difference between during and after spreading within each type of manure.

The emission rates of total bacteria statistically varied between the different types of manure (*p* < 0.05, Figure 2). Specifically, the median rates during spreading were the highest for poultry manure (1.07 × 10^11^ gene copies/kg of manure spread), followed by cow manure (5.85 × 10^9^ gene copies/kg), pig slurry with the dribble bar (3.99 × 10^9^ gene copies/kg of manure spread), and pig slurry with the splash plate (2.24 × 10^7^ gene copies/kg). Overall, the emission rates of total bacteria appeared to be higher during spreading compared to after, but no statistical difference was observed.

Although we used a dust track to measure different particulate matters, unfortunately, the results did not lead to any interpretable results, as in most cases the airborne dust particles returned to normal levels shortly after manure application, and the results did not reveal any significant differences.

### 3.3. Airborne Emissions of Fecal Indicators

*Archaea* emission rates were statistically different between manure types (*p* < 0.05, Figure 2). The reported median rates during spreading were the highest for poultry manure (2.89 × 10^6^ 16S rRNA gene copies/kg), followed by both pig slurry with the splash plate (4.07 × 10^5^ 16S rRNA gene copies/kg) and pig slurry with the dribble bar (3.06 × 10^5^ 16S rRNA gene copies/kg), as well as cow manure (2.12 × 10^5^ 16S rRNA gene copies/kg). Again, no difference could be observed between “during spreading” and “after spreading”, although values tended to be higher during spreading regardless of the type of manure spread.

*E. coli* emission rates significantly differed between different types of manure (*p* < 0.05, Figure 2). As for *E. coli*, during spreading, the median rates were once again the highest for poultry manure (1.88 × 10^4^ 16S rRNA gene copies/kg), followed by cow manure (6.46 × 10^3^ 16S rRNA gene copies/kg), pig slurry with the splash plate (5.97 × 10^2^ 16S rRNA gene copies/kg), and pig slurry with the dribble bar (5.22 × 10^2^ 16S rRNA gene copies/kg). Moreover, no statistical difference was observed between “during spreading” and “after spreading” samples for *E. coli* and *Archaea,* while emission rates for *Enterococcus* decreased immediately after manure spreading for both poultry manure and pig slurry with the splash plate (*p* < 0.05). That was not the case for cow manure or pig slurry with the dribble bar. Eventually, no statistical difference was observed within different manure types regarding the during and after time periods for *Aerococcus* phage (Figure S3).

### 3.4. Antibiotic Resistance Genes’ (ARGs) Emission Rates

**Figure 3:**
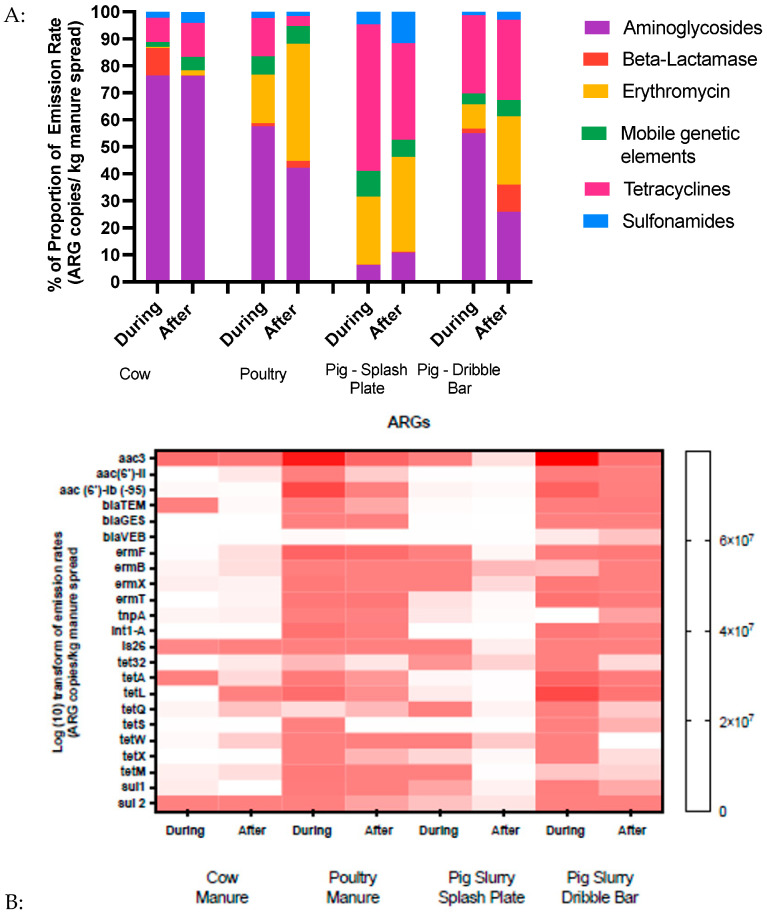
Emission rates of ARGs in the air. (**A**) Proportion of emission rates of total ARGs in the air. (**B**) Heatmap illustrating emission rates of different ARGs in the air. The colors in the heat map represent the log-fold changes in emission rates, with white indicating low upregulation and red indicating higher upregulation.

Patterns of airborne antimicrobial resistance genes significantly differed between the different types of manure (Figure 3A). More specifically, the relative abundance of aminoglycoside resistance genes was found to be the most abundant in bioaerosols associated with cow manure spreading, reaching ~80% of the total ARGs in terms of concentration. In comparison, they represented less than 60% of the total ARGs for the other types of manure and even dropped down to less than 10% in the pig slurry with splash plate bioaerosols. However, the lowest concentrations of total airborne ARGs were found in cow manure, whether during spreading (1.23 × 10^6^ gene copies/m^3^) or after (7.98 × 10^5^ genes copies/m^3^) (Figure 3B and Table 2).

Interestingly, the concentration and relative contribution of airborne ARGs strongly differed between the two methods of spreading of the pig slurry, despite that they were taken from the same farm (Figure 3A). Specifically, tetracycline resistance largely dominated in the air during the spreading when using the splash plate, reaching ~55% of total ARGs, but was but less than ~30% when using the dribble bar. Conversely, aminoglycoside resistance represented ~55% of total ARGs during the spreading of pig slurry when using the dribble bar but was less than 10% when using the splash plate. Additionally, except for MGEs and sulfonamides, the emission rates of almost all groups of ARGs, including resistance for aminoglycosides, beta-lactams, and erythromycin, were significantly different when comparing pig slurry with the dribble bar and pig slurry with the splash plate (*p* < 0.05). The specific *aac (6)*–*lb*, *blaGES*, and *blaVEB* resistance genes stayed constant during and after spreading for both the pig slurry splash plate and dribble bar experiments (Figure 3B).

Similar to cow manure, aminoglycosides resistance-encoding genes represented majority of the detected airborne ARGs for poultry manure (~55%, Figure 2A). However, the lowest concentrations of airborne total ARGs could be observed compared to other types of manure, with levels of 4.91 × 10^6^ copies of genes/m^3^ during spreading and 1.31 × 10^6^ copies of genes/m^3^ after spreading (Table 2 and Figure 3B).

The results also revealed that the emission rates of aminoglycoside resistance genes were significantly different between cow manure and pig slurry with the splash plate (*p* < 0.05). In the case of erythromycin resistance genes, apart from cow manure vs. pig slurry with the splash plate and pig slurry with the dribble bar vs. poultry manure, all other manure types showed a significantly different pattern (*p* < 0.05). The emission rates for mobile genetic elements also showed a significant difference between pig slurry with the splash plate and poultry manure (*p* < 0.05). No other significant difference was observed among different groups of manure for tetracycline and sulfonamide resistance genes.

## 4. Discussion

Our study investigated the impact of manure spreading on airborne bacterial emissions and antibiotic resistance genes, providing valuable insights into the differences in emission rates among manure types and spreading methods. These findings emphasize the need for effective management strategies to minimize the spread of antibiotic resistance genes and reduce the potential health risks associated with manure application. While there are other studies investigating the impact of manure spreading on airborne bacterial emissions and antibiotic resistance genes, our study is original in its controlled setting, the use of different manure types and spreading methods, and in the quantification of various types of bacteria, and ARGs. Our findings contribute to the existing literature on this topic and highlight the importance of manure spreading in risk assessments of human and animal health.

In the present work, we found that the spreading of manure can be associated with significant emissions of total bacteria, *Archaea*, *Enterococcus,* and *E. coli* in the air, as previously reported [24]. Here, the different types of manure (poultry, cow, and pig slurry) and associated spreading methods (with the splash plate or dribble bar) resulted in very different emission rates. This is consistent with previous studies that have found differences in the airborne emissions of various compounds from different types of manure [25]. Usually, we expect airborne emissions of bioaerosols to be tightly linked with their related concentration in its source (manure). Here, the emission rates of total bacteria, *Archaea,* and *E. coli* were the highest during poultry manure spreading, whose measured concentrations in manure were lower than those observed for cow manure and pig slurry. These observed differences can be attributed to a number of factors, including differences in the microbial communities in the manure, the physicochemical properties of the manure (e.g., dry matter content, granulometry, or particle size distribution), and the management practices used for handling and spreading the manure [26,27,28]. Interestingly, *Archaea* were found in the manure in high abundances. This is consistent with their reported presence in various animal wastes, such as pig slurry, cow manure, and human feces [29], but differs from their proportions in the intestinal and gut microbiota of animals compared to bacteria. The resilience of the anaerobic co-digestion process in dairy and poultry waste has been shown to be attributed to the vigorous *Archaeal* microbial population, which demonstrated remarkable stability despite the rise in ammonia levels and the organic loading rate [30]. These findings suggest that *Archaea* may have important implications for the management of animal waste and the environmental impact of animal farming. Additionally, these *Archaea* could be released into the air, as bioaerosols can be found in significant quantities in working environments such as farms, wastewater treatment plants, and other similar facilities. Moreover, the presence of up to 10^8^ bacteria and 10^6^ *Archaea*/m^3^ of air was observed in dairy farms and poultry production environments [31]. However, airborne *Archaea* have received relatively little attention, and therefore, the understanding of these microorganisms remains limited.

Manure contains important quantities of ARGs (Table 2) carried by bacteria; therefore, the emission rates of airborne ARGs were specifically measured. We found that aminoglycosides, tetracyclines, and erythromycins were the most abundant in the air following the spreading of manure, and that these are not necessarily related to animal care. For example, beef and dairy cattle are systematically treated with chlortetracycline, tylosin, and sulfamethoxazole [32]. The emission rates of airborne aminoglycoside resistance genes were higher for cow manure and for pig slurry spread with dribble bars. Beta-lactamase genes and MGEs were only higher in pig slurry with the dribble bar when compared to pig slurry with the splash plate. Cow manure emitted fewer erythromycin resistance gene copies than poultry manure and pig slurry with the dribble bar, while poultry manure emitted fewer erythromycin resistance genes than pig slurry with the splash plate, and pig slurry with the splash plate emitted fewer genes than pig slurry with the dribble bar.

In poultry, tetracycline, tylosin, salinomycin, bambermycin, penicillin, and trimethoprim-sulfonamides are commonly used antibiotics in North America [33]. Moreover, in pigs, the most used antibiotics are tylosin, chlortetracycline, bacitracin, and lincomycin [34]. Therefore, ARGs associated with these antibiotics may also repeatedly appear in both manure and air samples. For instance, *tetX* has appeared to be the most abundant ARG in sheep, poultry, and cow manure samples in China [35]. More studies [36] have revealed that the level of some ARGs and MGEs may decrease during the composting procedure of manure. These results indicated a drop in the levels of *sul2* as well as *ermA, ermB, ermF,* and *ermX* genes in composted manure [36]. The presence of these antibiotics (or ARGs) can be harmful for humans and animals. Further, aminoglycoside resistance genes have been reported in high concentrations in bioaerosol samples collected from pig buildings using next-generation, high-throughput sequencing approaches [37]. This could explain why the concentrations of some ARGs in manure differ from what was expected. These results are in line with our study, showing a lower risk for cow manure in terms of erythromycin resistance genes, whose ARG concentrations were the lowest (1.23 × 10^6^ gene copies/m^3^ manure spread).

It is noteworthy that the emission of different ARGs rapidly went back to normal after the spreading. For instance, emissions for aminoglycosides in poultry and pig slurry with the dribble bar went back to normal after spreading. However, cow manure and pig slurry with the splash plate showed different patterns for the same group of antibiotics. Conversely, ARGs associated with erythromycin never showed a pattern for any groups of antibiotics and never went back to normal after spreading. This could be dependent on several factors, such as the wind direction and the fact that erythromycin could have already existed in the environment. Further, tetracycline resistance genes went back to normal in poultry and pig slurry with the splash plate experiments, while these emissions were constant for cow manure and pig slurry with the dribble bar.

Our findings indicate a statistically significant difference in the emissions of *Enterococcus* during and after the spreading manure. Specifically, higher emissions during the spreading were noted for poultry manure, and pig slurry with the splash plate. However, no difference was observed for cow manure or pig slurry with the dribble bar, which could mean that after spreading, *Enterococcus* may have settled onto the ground or been dispersed by the wind, leading to lower emission rates for the latter manure types [38]. Additionally, *Aerococcus* phage was found at high concentrations in pig slurry (10^6^ to 10^7^ gene copies/g dry matter), poultry manure (10^5^ to 10^6^ gene copies/g dry matter), and cow manure (10^3^ to 10^4^ gene copies/g dry matter), highly suggesting the presence of the related host. They were not detected in air samples.

Considering the volume of manure spread each year, in the Province of Quebec, that is around 10^9^ kg for swine manure [39] and given the average total bacterial emission obtained in our experimental setup (10^9^ 16S copies/kg of manure spread), we can estimate the total number of bacteria emitted in the air during swine manure-spreading activities to be up to 10^18^ per year. This gives a reference of the importance of this activity in the overall bacteria emissions in the air and the potential for human and animal exposure to antibiotic resistance genes. Moreover, this suggests that exposure to high levels of bacteria from manure emissions can pose a significant risk to human health, including respiratory and gastrointestinal infections, particularly when working with or living near livestock operations [24]. However, it is important to note that the presence and concentrations of specific species of bacteria in each type of manure can also affect the level of risk. For example, bacteria commonly found in manure, such as *E. coli* and *Archaea,* are responsible for a wide range of illnesses in humans, including gastrointestinal infections, pneumonia, and meningitis [40,41,42]. In addition, exposure to antibiotic-resistant bacteria can increase the risk of developing antibiotic-resistant and life-threatening infections, which are becoming an increasingly serious public health concern [43].

## 5. Conclusions

In conclusion, this study found that the emission rates of airborne total bacteria, *E. coli*, and *Archaea* significantly varied among the different types of manure and spreaders tested. Poultry manure had the highest emission rates of total bacteria, *E. coli*, and *Archaea*, followed by cow manure, pig slurry with the splash plate, and pig slurry with the dribble bar. Exposure to high levels of bacteria from manure emissions can pose a significant risk to human health, particularly to those working with or living near livestock operations. In the context of ARGs, the results suggested that the spreading of cow manure and pig slurry with the dribble bar was associated with higher emission rates of airborne aminoglycoside resistance genes. Furthermore, pig slurry spreading was linked to the highest rates of airborne erythromycin-resistant genes, followed by poultry and cow manure spreading. These findings highlight the potential environmental risks associated with manure spreading and the need for effective management strategies to minimize the spread of antibiotic resistance genes. Additionally, in most cases, the airborne dust particles returned to normal levels shortly after manure application, and the results did not reveal any significant differences. To sum up, this study highlighted the importance of managing manure application to minimize the emissions of airborne bacteria, ARGs, and reduce the potential health risks associated with exposure to airborne pollutants. Overall, manure spreading is emitting significant amounts of bioaerosols and antibiotic resistance genes and should be considered in overall human risk assessments.

## Figures and Tables

**Table 1:** Average concentrations of fecal indicators and ARGs in manure or different manure types and spreaders (16S rRNA gene copies/g of dry matter content). (A) Average concentration of total bacteria, fecal indicators, and Aerococcus phage in manure. (B) Average concentration of ARGs within different groups of antibiotics in manure.

(A)				
Gene Copies/g Dry Matter of Manure				
	Cow Manure	Poultry Manure	Pig Slurry with Splash Plate	Pig Slurry with Dribble Bar
Total bacteria	7.61 × 10^13^	5.70 × 10^11^	9.29 × 10^13^	1.91 × 10^13^
*Enterococcus*	1.91 × 10^7^	4.86 × 10^9^	2.75 × 10^9^	2.09 × 10^9^
*E. coli*	7.65 × 10^4^	1.19 × 10^8^	1.43 × 10^8^	1.19 × 10^8^
*Archaea*	2.54 × 10^10^	4.49 × 10^8^	2.39 × 10^10^	1.83 × 10^10^
*Aerococcus* Phage	1.45 × 10^4^	1.63 × 10^6^	1.74 × 10^7^	9.06 × 10^6^
**(B)**				
**Resistance Gene Copies/g Dry Matter of Manure**				
	**Cow Manure**	**Poultry Manure**	**Pig Slurry with Splash Plate**	**Pig Slurry with Dribble Bar**
Aminoglycosides	7.15 × 10^7^	1.71 × 10^7^	4.84 × 10^9^	1.6 × 10^9^
Beta-Lactamase	1.64 × 10^9^	4.69 × 10^9^	4.39 × 10^10^	1.70 × 10^13^
Erythromycin	2.77 × 10^10^	3.52 × 10^15^	6.72 × 10^15^	1.01 × 10^9^
MGE	5.61 × 10^9^	9.33 × 10^5^	4.34 × 10^11^	2.01 × 10^6^
Tetracycline	2.20 × 10^9^	1.34 × 10^10^	2.31 × 10^12^	1.42 × 10^12^
Sulfonamide	1.52 × 10^10^	7.04 × 10^9^	5.77 × 10^11^	2.75 × 10^11^
Quinolones	3.27 × 10^10^	1.61 × 10^8^	5.38 × 10^10^	6.50 × 10^9^
Vancomycin	2.6 × 10^7^	2.67 × 10^7^	1.11 × 10^8^	2.55 × 10^7^

**Table 2:** Absolute number of total ARGs in the air for different manure types and spreaders used for the sampling periods “during spreading” and “after spreading”.

	Absolute Number of Total ARGs in the Air during Spreading (Gene Copies/m^3^)	Absolute Number of Total ARGs in the Air after Spreading (Gene Copies/m^3^)
*Cow manure*	1.23 × 10^6^	7.98 × 10^5^
*Poultry manure*	4.91 × 10^6^	1.31 × 10^6^
*Pig slurry with splash plate*	1.23 × 10^6^	1.25 × 10^5^
*Pig slurry with dribble bar*	3.33 × 10^7^	7.18 × 10^6^

## Data Availability

Supporting data are available from the authors if requested.

## Supplementary Materials

The following supporting information can be downloaded at: https://www.mdpi.com/article/10.3390/microorganisms11071797/s1, Figure S1: (A) Positioning of the exhaust fans of the wind tunnel, and (B) a view of the wind tunnel and the downwind sampling site. Figure S2: Pictures of the different spreaders used to apply different types of manure inside the wind tunnel. The splash plate (A,D) and the dribble bar (E) were connected through a hose to a storage tank (B) and used to spread pig slurry. The horizontal beater spreader (C) was used to apply solid (poultry and cow) manure. Table S1: Physical characteristics of manure. Figure S3: Emission rate of the phage *vB_AviM_AVP* of *Aerococcus viridans* for the tested manure types. No difference was observed among the paired groups regarding the emission rates of the phage. Table S2: qPCR thermoprotocols. Table S3: Sequences of primers and probes used for quantification by PCR of total bacteria [44,45], *E. coli, Enterococcus* [46], *Archaea* [47,48], the phage *vB_AviM_AVP* of *Aerococcus viridans*, ARGs, and MGEs [22]. Table S4: Insert sequences used to construct standard curves that were used for qPCR detection of targeted ARGs and MGEs.
