# Correction: Baghdadi et al. Contribution of Manure-Spreading Operations to Bioaerosols and Antibiotic Resistance Genes’ Emission. *Microorganisms* 2023, *11*, 1797

**DOI:** 10.3390/microorganisms13030625

**Published:** 2025-03-10

**Authors:** Mahsa Baghdadi, Patrick Brassard, Stéphane Godbout, Valérie Létourneau, Nathalie Turgeon, Florent Rossi, Émie Lachance, Marc Veillette, Marie-Lou Gaucher, Caroline Duchaine

**Affiliations:** 1 Département de Biochimie, de Microbiologie et de Bio-Informatique, Faculté des Sciences et de Génie, Université Laval, Québec, QC G1V 0A6, Canada; mahsa.baghdadi.1@ulaval.ca (M.B.);; 2 Centre de Recherche de L’institut de Cardiologie et de Pneumologie de Québec, Québec, QC G1V 4G5, Canada; 3 Institut de Recherche et de Développement en Agroenvironnement, Québec, QC G1P 3W8, Canada; 4 Chaire de Recherche en Salubrité des Viandes, Département de Pathologie et Microbiologie, Faculté de Médecine Vétérinaire, Université de Montréal, Saint-Hyacinthe, QC J2S 2M2, Canada; 5 Canada Research Chair on Bioaerosols, Québec, QC G1V 4G5, Canada

In the original publication [1], there was a mistake in Table 1 legend as published. The mistake involved missing “Average concentrations” in the legend. The miscalculation involved applying the equation (−0.3082 × 10^8^) + 12.223 to data where some values were zero, leading to incorrect results. The corrected Table 1 appears below. 

In the original publication, there was a mistake in the Figure 1 caption as published. The mistake involved a typo in “ARGs”, which was edited to ARG. The corrected Figure 1 appears below

In the Figure 3A legends, Sulfanamide is a typo and needs to be changed to sulfonamides. The corrected Figure 3A appears below. 

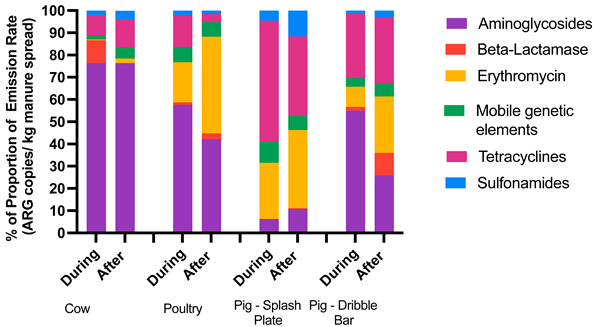



The miscalculation involved applying the equation (−0.3082 × 10^8^) + 12.223 to data where some values were zero, leading to incorrect results. This error resulted in errors in the descriptions of Table 1 and Figure 1 and the related text. A correction has been made to the results Section 3.1. The corrected section appears below. 

“Manure is known as the main source of emitted bioaerosols. In the present study, the average concentration of total bacteria was found to be the highest in cow manure and pig slurry (7.61 × 10^13^, 9.29 × 10^13^, and 1.91 × 10^13^ 16S rRNA gene copies/g of dry matter, respectively) and the lowest in poultry manure (Table 1A). A similar observation can be made for *Archaea*, which was 2 log lower in the latter. Conversely, *Enterococcus*, *E. coli*, and the *Aerococcus* phage were the lowest in cow manure (1.91 × 10^7^, 7.65 × 10^4^, and 1.45 × 10^4^ 16S rRNA gene copies/g of dry matter, respectively), whereas the other types of manure displayed very similar concentrations (Table 1A).

Beta-lactamase and tetracyclines, as well as sulfonamides, resistance genes were the most abundant ARGs in pig slurry with the dribble bar (1.70 × 10^13^, 1.42 × 10^12^, and 2.75 × 10^11^ gene copies/g of dry matter, respectively) (Table 1B). Erythromycin and tetracycline resistance genes were the most reported ARGs in pig slurry with the splash plate (6.72 × 10^15^, 2.31 × 10^12^, gene copies/g of dry matter, respectively). Erythromycin resistance genes had the highest concentration in poultry manure (3.52 × 10^15^ gene copies/g of dry matter). According to these results, on average, erythromycin and beta-lactamase resistance genes were the most abundant in manure for most of the experiments (10^15^ and 10^12^ gene copies/g of dry matter) (Figure 1).”

The authors state that the scientific conclusions are unaffected. This correction was approved by the Academic Editor. The original publication has also been updated. 

## Figures and Tables

**Figure 1:**
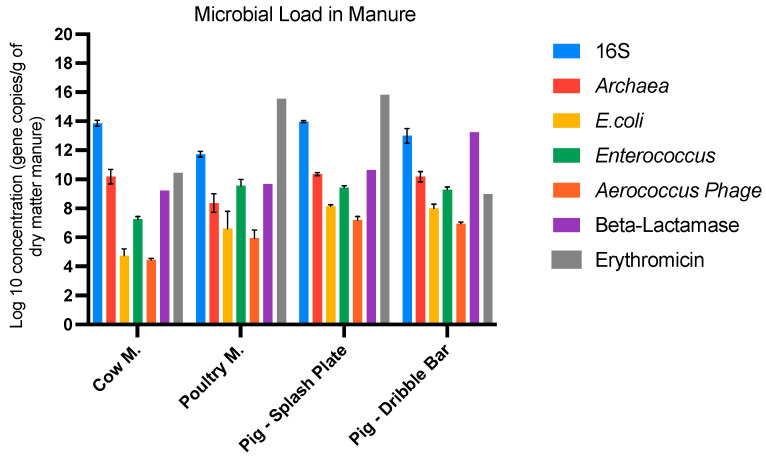
Concentration of total bacteria, fecal indicators, phage, and the most abundant ARG in the manure.

**Table 1:** Average concentrations of fecal indicators and ARGs in manure or different manure types and spreaders (16S rRNA gene copies/g of dry matter content). (A) Average concentration of total bacteria, fecal indicators, and Aerococcus phage in manure. (B) Average concentration of ARGs within different groups of antibiotics in manure.

(A)				
Gene Copies/g Dry Matter of Manure				
	Cow Manure	Poultry Manure	Pig Slurry with Splash Plate	Pig Slurry with Dribble Bar
Total bacteria	7.61 × 10^13^	5.70 × 10^11^	9.29 × 10^13^	1.91 × 10^13^
*Enterococcus*	1.91 × 10^7^	4.86 × 10^9^	2.75 × 10^9^	2.09 × 10^9^
*E. coli*	7.65 × 10^4^	1.19 × 10^8^	1.43 × 10^8^	1.19 × 10^8^
*Archaea*	2.54 × 10^10^	4.49 × 10^8^	2.39 × 10^10^	1.83 × 10^10^
*Aerococcus* Phage	1.45 × 10^4^	1.63 × 10^6^	1.74 × 10^7^	9.06 × 10^6^
**(B)**				
**Resistance Gene Copies/g Dry Matter of Manure**				
	**Cow Manure**	**Poultry Manure**	**Pig Slurry with Splash Plate**	**Pig Slurry with Dribble Bar**
Aminoglycosides	7.15 × 10^7^	1.71 × 10^7^	4.84 × 10^9^	1.6 × 10^9^
Beta-Lactamase	1.64 × 10^9^	4.69 × 10^9^	4.39 × 10^10^	1.70 × 10^13^
Erythromycin	2.77 × 10^10^	3.52 × 10^15^	6.72 × 10^15^	1.01 × 10^9^
MGE	5.61 × 10^9^	9.33 × 10^5^	4.34 × 10^11^	2.01 × 10^6^
Tetracycline	2.20 × 10^9^	1.34 × 10^10^	2.31 × 10^12^	1.42 × 10^12^
Sulfonamide	1.52 × 10^10^	7.04 × 10^9^	5.77 × 10^11^	2.75 × 10^11^
Quinolones	3.27 × 10^10^	1.61 × 10^8^	5.38 × 10^10^	6.50 × 10^9^
Vancomycin	2.6 × 10^7^	2.67 × 10^7^	1.11 × 10^8^	2.55 × 10^7^
