# Supplementary material for: Contribution of Manure-Spreading Operations to Bioaerosols and Antibiotic Resistance Genes’ Emission
Source: Microorganisms. 2023 Jul 13;11(7):1797. doi: 10.3390/microorganisms11071797 (PMC10386661; doi:10.3390/microorganisms11071797)
Supplement: Supplementary file 1 [file microorganisms-11-01797-s001.zip › microorganisms-2476752-supplementary.pdf]

Contribution of Manure-Spreading Operations to Bioaerosols and Antibiotic Resistance Genes' Emission

Mahsa Baghdadi [1,2], Patrick Brassard [3], Stéphane Godbout [3], Valérie Létourneau [1,2], Nathalie Turgeon [1,2], Florent Rossi [1,2], Émie Lachance [1,2], Marc Veillette [1,2], Marie-Lou Gaucher [4] and Caroline Duchaine [1,2,5,*]

Supplementary Material

Section S1. Wind tunnel characteristics

A field-scale wind tunnel was built at IRDA (*Institut de recherche et de développement en agroenvironnement, Québec, Canada*) to spread manure in a controlled environment and measure gas, odor and bioaerosol emissions. The wind tunnel was scaled to accommodate agricultural machinery used for small-scale farming operations (8 m width x 30 m length x 4 m height). Air was drawn into the wind tunnel by ten, speed tunable, 24-inch diameter fans who allow wind speed of 0.2 to 1.2 m/s (5 to 30 m^3/s) The environmental conditions (wind speed, temperature, and humidity) are closely monitored in the tunnel with an INTERCAP® humidity and temperature probe (VAISALA - HMP60). The speed of the fans was always 0.32 m/s which is equal to 8 m^3/s in this experiment [20]. Inside the wind tunnel, an (8 m width x 22 m length x 0.6 height) strip of soil harvested from the nearby farming sites was disposed. The wind tunnel is accessible by two doors (front and back). Spreader-vehicles' entrance was located at the back of the tunnel (upwind) and spreading was always performed from the back to the front of the tunnel (downwind to upwind) by operating two successive back and forth on the strip of soil for solid manure, and only one pass for pig slurry. All doors were closed during spreading. Because the strip of soil tends dry quickly inside the wind tunnel, the soil was irrigated before each spreading on the entire surface to prevent artificial particle emissions caused by the passage of the spreader-vehicle.

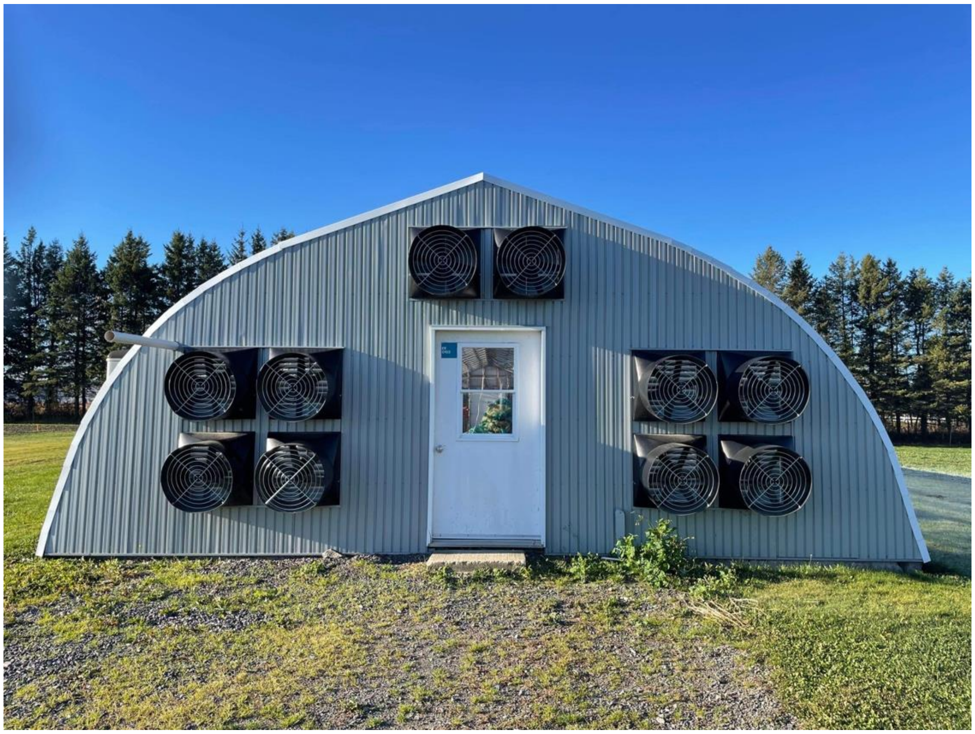

A.

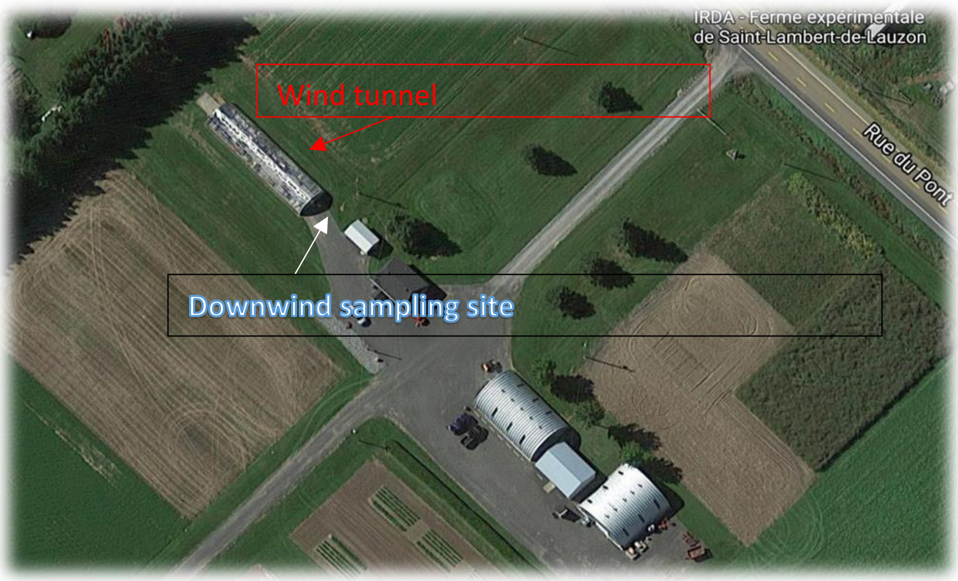

B.

Figure S1. A: Positioning of the exhaust fans of the wind tunnel and **B:** a view of wind tunnel and the downwind sampling site

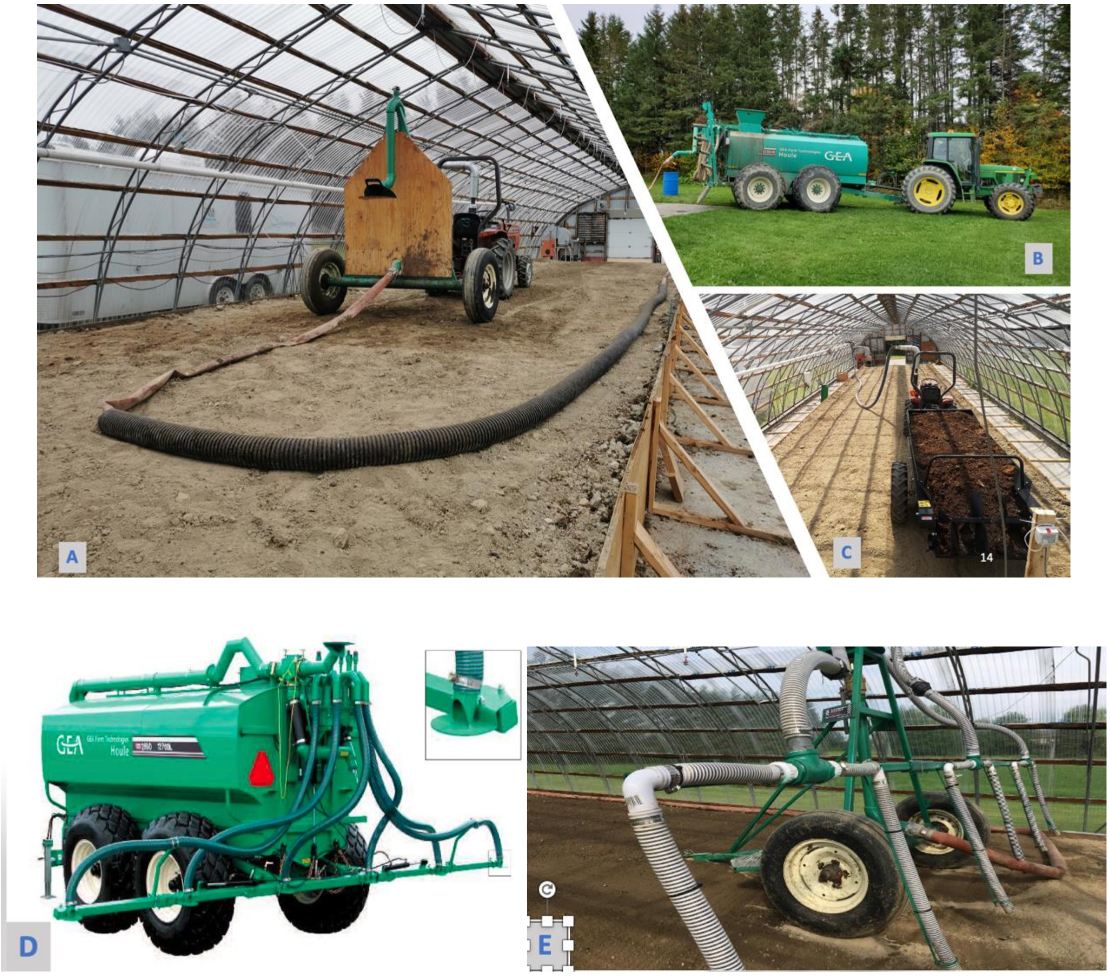

Figure S2. Pictures of the different spreaders used to apply different types of manure inside the wind tunnel. Splash plate (A, D) and dribble bar (E) were connected through a hose to a storage tank (B) and used to spread pig slurry. Horizontal beater spreader (C) was used to apply solid (poultry and cow) manure.

Table S1. Physical characteristics of manure

	Number of samplings	Dry Matter content (%)
Cow Manure	Field trip 6	36.5
	Field trip 7	34.8
	Field trip 8	29.3
	Field trip 9	35.4
	Field trip 10	39.4
	Field trip 11	34.5
Pig slurry - Splash plate	Field trip 12	8.89
	Field trip 13	7.90
	Field trip 14	7.91
	Field trip 15	9.04
	Field trip 16	8.47
	Field trip 17	8.55
Poultry Manure	Field trip 18	47.9
	Field trip 19	48.8
	Field trip 20	59.6
	Field trip 21	54.3
	Field trip 22	50.5
	Field trip 23	53.7
Pig slurry - Drible Bar	Field trip 24	6.67
	Field trip 25	6.47
	Field trip 26	6.16
	Field trip 27	6.44
	Field trip 28	11.2
	Field trip 29	11.2

Table S2. qPCR thermoprotocols

qPCR Protocols	Total bacteria (EUB)	40 cycles
**Thermo Protocol**	95 °C	3 min
	95 °C	20s
	62 °C	1 min
	Go to step 2	39x
	***Aeroccocus* Phage**	**40 cycles**
**Thermo Protocol**	95 °C	3 min
	95 °C	15s
	60ºC	60s
	Go to step 2	39x
	Archaea- rRNA 16S	**40 cycles**
**Thermo Protocol**	95 °C	3 min
	95 °C	10s
	55.5 °C	20s
	72 °C	25s
	Go to step 2	39X
	72 °C	10 min
**Melting Curve**	50 °C	31s
	50 °C	0.02 + 0.5/s
	Go to step 9	90X
	***Entero- rRNA* 16S**	**40 cycles**
**Thermo Protocol**	95 °C	3 min
	94 °C	15s
	57 °C	30s
	72 °C	30s
	Go to step 2	39X
	72 °C	10 min
**Melting Curve**	50 °C	31s
	50 °C	0.02 + 0.5/s
	Go to step 9	90X
	***E.coli- rRNA* 16S**	**40 cycles**
**Thermo Protocol**	95 °C	3 min
	95 °C	15s
	55 °C	30s
	72 °C	30s

	Go to step 2	39X
	72 °C	10 min
**Melting Curve**	50 °C	31s
	50 °C	0.02 + 0.5/s
	Go to step 9	90X

Table S1. Sequences of primers and probes used for quantification by PCR of total bacteria [44,45], *E. coli* , *Enterococcus* [46], *Archaea* [47,48], the phage *vB_AviM_AVP* of *Aerococcus viridans*, ARGs and MGEs [22].

**Targeted microorganisms or antibiotic resistances**	**Targeted genes**	**Sequence of primers and probes**
Total bacteria	16S rRNA	Forward:GACARCCATGCASCACCTG<br>Reverse:GGTAGTCYAYGCMSTAAACG<br>Probe: FAM/TKC GCG TTG/ZEN/CDT CGA ATT AAW CCA C/3IABkFQ
Enterococcus	16S rRNA	Forward : CCCTTATTGTTAGTTGCCATCATT<br>Reverse : ACTCGTTGTACTTCCCATTGT
E. coli	16S rRNA	Forward : GTTAATACCTTTGCTCATTGA<br>Reverse : ACCAGGGTATCTAATCCTGTT
Archaea	16S rRNA	Forward : CCGACGGTGAGRGRYGAA<br>Reverse : YCCGGCGTTGAMTCCAAT T
Phage vB_AviM_AVP of *Aerococcus viridans*	DNA polymerase	Forward : CTACACAGACATGGGYGGATATG<br>Reverse : CTACACAGACATGGGYGGATATG<br>Probe: TGATGCCTTAGAGGACTACAAGAAGA
Aminoglycoside resistance	aac(6')-II (8)	Forward : CGACCCGACTCCGAACAA<br>Reverse: GCACGAATCCTGCCTTCTCA
Aminoglycoside resistance	aac(6')-Ib (95)	Forward: CGTCGCCGAGCAACTTG<br>Reverse: CGGTACCTTGCCTCTCAAACC
Aminoglycoside resistance	aac(3)-iid_iii_iif_iia_iie (410)	Forward:<br>CGATGGTCGCGGTTGGTC<br>Reverse:<br>TCGGCGTAGTGCAATGCG
Beta-Lactamaase resistance	blaCMY2 (108)	Forward: AAAGCCTCATGGGTGCATAAA<br>Reverse: ATAGCTTTTGTTTGCCAGCATCA
Beta-Lactamaase resistance	blaGES (120)	Forward: GCAATGTGCTCAACGTTCAAG<br>Reverse: GTGCCTGAGTCAATTCTTTCAAAG
Beta-Lactamaase resistance	blaVEB (38)	Forward: CCCGATGCAAAGCGTTATG<br>Reverse: GAAAGATTCCCTTTATCTATCTCAGACAA
Beta-Lactamaase resistance	blaTEM (164)	Forward: AGCATCTTACGGATGGCATGA<br>Reverse: TCCTCCGATCGTTGTCAGAAGT
Beta-Lactamaase resistance	blaVIM (147)	Forward:<br>GCACTTCTCGCGGAGATTG<br>Reverse :<br>CGACGGTGATGCGTACGTT
Beta-Lactamaase resistance	blaIMP (324)	Forward:<br>GGAATAGAGTGGCTTAATTC<br>Reverse:<br>GGTTTAACAAAACAACCACC
Beta-Lactamaase resistance	blaMOX (34)	Forward: CTATGTCAATGTGCCGAAGCA<br>Reverse: GGCTTGTCCTCTTTCGAATAGC
Beta-Lactamaase resistance	blaSHVII (1110)	Forward: TTGACCGCTGGGAAACGG<br>Reverse: TCCGGTCTTATCGGCGATAAAC

Beta-Lactamaase resistance	blaOXA (1506)	Forward:CGCAATTATCGGCCTA GAAACT Reverse: TTGGCTTTCCGTCCCATTT
Beta-Lactamaase resistance	blaCTX-M-1	Forward: CGG GCR ATG GCG CAR AC Reverse : TGC RCC GGT SGT ATT GCC Probe: FAM/CCA RCG GGC/ZEN/GCA GYT GGT GAC/3IABkFQ
Erythromycine resistance	ermB (804)	Forward: GAACACTAGGGTTGTTCTTGCA Reverse: CTGGAACATCTGTGGTATGGC
Erythromycine resistance	ermF (23)	Forward: CAGCTTTGGTTGAACATTTACGAA Reverse: AAATTCCTAAAATCACAACCGACAA
Erythromycine resistance	ermT (137)	Forward:GTTCACTAGCACTATTTTTAATGACA GAAGT Reverse: GAAGGGTGTCTTTTTAATACAATTAACGA
Erythromycine resistance	ermX (209)	Forward:GCTCAGTGGTCCC CATGGT Reverse: ATCCCCCCGTCAACGTT
Erythromycine resistance	erm35 (815)	Forward: CCTTCAGTCAGAACCGGCAA Reverse: GCTGATTTGACAGTTGGTGGTG
Tetracycline resistance	tet32 (54)	Forward: CCATTACTTCGGACAACGGTAGA Reverse: CAATCTCTGTGAGGGCATTTAACA
Tetracycline resistance	tetA (180)	Forward: CTCACCAGCCTGACCTCGAT Reverse: CACGTTGTTATAGAAGCCGCATAG
Tetracycline resistance	tetL (195)	Forward:ATGGTTGTAGTTGCGCG CTATAT Reverse: ATCGCTGGACCGACTCCTT
Tetracycline resistance	tetO (192)	Forward :CAACATTAACGGAAAGTTTATTG TATACCA Reverse: TTGACGCTCCAAATTCATTGTATC
Tetracycline resistance	tetQ (185)	Forward:CGCCTCAGAAGTAAGTTCATAC ACTAAG Reverse: TCGTTCATGCGGATATTATCAGAAT
Tetracycline resistance	tetS (200)	Forward:TTAAGGACAAACTTTCTGAC GACATC Reverse: TGTCTCCCATTGTTCTGGTTCA
Tetracycline resistance	tetW (191)	Forward: ATGAACATTCCCACCGTTATCTTT Reverse: ATATCGGCGGAGAGCTTATCC
Tetracycline resistance	tetX (196)	Forward: AAATTTGTTACCGACACGGAAGTT Reverse: CATAGCTGAAAAAATCCAGGACAGTT
Tetracycline resistance	tetM (1513)	Forward: GGAGCGATTACAGAATTAGGAAG C Reverse: TCCATATGTCCTGGCGTGTC
Vancomycin resistance	vanA (1514)	Forward: GGGCTGTGAGGTCGGTTG Reverse: TTCAGTACAATGCGGCCGTTA
Vancomycin resistance	vanB (211)	Forward: TTGTCGGCGAAGTGGATCA Reverse: AGCCTTTTTCCGGCTCGTT
Vancomycin resistance	vanRA (216)	Forward: CCCTTACTCCCACCGAGTTTT Reverse: TTCGTCGCCCCATATCTCAT
Vancomycin resistance	vanSA (218)	Forward: CGCGTCATGCTTTCAAAATTC Reverse: TCCGCAGAAAGCTCAATTTGTT
Sulfonamide resistance	sul1 (363)	Forward: GCCGATGAGATCAGACGTATT G Reverse: CGCATAGCGCTGGGTTTC

Sulfonamide resistance	sul2 (133)	Forward:<br>TCATCTGCCAAACTCGTCGTTA<br>Reverse:<br>GTCAAAGAACGCCGCAATGT
Quinolone resistance	qnrB (1202)	Forward: TCACCACCCGCACCTG<br>Reverse: GGATATCTAAATCGCCCAGTTCC
Colistine resistance	mcr-1	Forward: ATGGCACGGTCTATGATA<br>Reverse: CGGATAATCCACCTTAACA<br>Probe: FAM/CTA CAG ACC/ZEN/GAC CAA GCC GA/3IABkFQ/-3'
Mobile Genetic Element resistance	is26 (1546)	Forward:<br>ATGGATGAAACCTACGTGAAGGTC<br>Reverse:<br>CGGTACTTAATCTGTCGGTGTTCA
Mobile Genetic Element resistance	tnpA (207)	Forward: AATTGATGCGGACGGCTTAA<br>Reverse: TCACCAAACTGTTTATGGAGTCGTT
Mobile Genetic Element resistance	int1-A (336)	Forward:<br>CGAAGTCGAGGCATTTCTGTC<br>Reverse:<br>GCCTTCCAGAAAACCGAGGA

Table S4. Insert sequencesused to construct standard curves that was used for qPCR detection of targeted ARGs and MGEs

Genes	Insert sequences
aac(6')-II	ATCTTCTCCCGCACGAATCCTGCCTTCTCATAGCAGCGTATGGCTCGATGGTTGTTCGGAGTCGGGTCGGTCTGAATCT
aac(6')-Ib	TTAGGCATCACTGCGTGTTCGCTCGAATGCCTGGCGTGTTTGAACCATGTACACGGCTGGACCATATGGGGTGGTTA
aac(3)- iid_iii_iif_iia_iie	CTAACCTGAAGGCTCGCAAGAGCGCTCGACGGCCTCGTGCGGAGGCACGATCGGAGTGGTTCCGAAATGCTTCTCAA<br>GATAGGTGACGCCGAACGTCACGATGTCCTGCGCGTCGAACAGGTAGCACTGAGCAAAGCCCACGACACCTTCTCGAT<br>GGCGACCGAGCTTCACGTAAGCATTTGCTATAGTTTCAACCGCATCCGGCTTTCCTTCGATAGCAAAGCAATCGAGAATG<br>CCGTTTGAATCGTAATCCGATGCCGTTTTCCAGGCGACTTCACCGTCTCTTCCAAGCATCGGCATCTCATACGTCACCCAC<br>CGTTTGTTGGGGATATCGGCAACCGCC
blaCMY2	CCCCGCAGTGAAAGCCTCATGGGTGCATAAAACGGGCTCCACTGGTGGATTTGGCAGCTACGTAGCCTTCGTTCCAGAAAAA<br>AACCTTGGCATCGTGATGCTGGCAAACAAAAGCTATCCTAACCCTG
blaGES	GCAGCGTTTTGCAATGTGCTCAACGTTCAAGTTTCCGCTAGCCGCGCTGGTCTTTGAAAGAATTGACTCAGGCACCGAGCGGGGG
blaVEB	ACTTCCATTTCCCGATGCAAAGCGTTATGAAATTTCCGATTGCTTTAGCCGTTTTGTCTGAGATAGATAAAGGGAATCTTTCTTTTGAACAA
blaTEM	GTCACAGAAAAGCATCTTACGGATGGCATGACAGTAAGAGAATTATGCAGTGCTGCCATAACCATGAGTGATAACACTGCTGCCAACTTAC<br>TTCTGACAACGATCGGAGGACCGAAGGAGC
blaVIM	ATGTTCAAACTTTTGAGTAAGTTATTGGTCTATTTGACCGCGTCTATCATGGCTATTGCGAGTCCGCTCGCTTTTTCCGTAGATTCTAGCGGTG<br>AGTATCCGACAGTCAGCGAAATTCCGGTCGGGGAGGTCCGGCTTTACCAGATTGCCGATGGTGTTTGGTCGCATATCGCAACGCAGTCGTTT<br>GATGGCGCAGTCTACCCGTCCAATGGTCTCATTGTCCGTGATGGTGATGAGTTGCTTTTGATTGATACAGCGTGGGGTGCGAAAAACACA<br>GCG
blaIMP	ATGAGCAAGTTATCTGTATTCTTTATATTTTTGTTTTGTAGCATTGCTACCGCAGCAGAGCCTTTGCCAGATTTAAAAATTGAAAAACTTGAT<br>GAAGGCGTTTATGTTCATACTTCGTTTGAAGAAGTTAACGGGTGGGGCGTTTTTCCTAAACATGGTTTGGTTGTTCTTGTAGATGCTGAAGCTTA<br>TCT<br>AATTGACACTCCATTTACGGCTAAAGATACTGAAAAGTTAGTCACTTGGTTTGTGGAACGTGGCTATAAAATAAAAGGCAGTATTTCCTCTC<br>ATTT<br>TCATAGTGACAGCACGGGC
blaMOX	ATGCAACAACGACAATCCATCCTGTGGGGCGCTCTGGCCACCCTGATGTGGGCCGGTCTGGCCCATGCAGGTGAGACTTCACCGGTCGATCCC<br>CTGCGCCCCGTGGTGGATGCCAGCATCCGGCCGCTGCTCAAGGAGCACAGGATCCCGGGCATGGCGGTGGCCGTGCTCAAGGATGGCAAGGC<br>CCACTATTTCAACTACGGTGTGGCCGATCGGGAGCGCGCAGTCGGTGTCAGCGAGCAGACCCTGTTCGAGATAGGCTCCGTGAGCAAGCCCCT<br>GACCGCGACCCTAGGAGCCTATGCGGTGGTCAAGGGAGCGATGCAACTGGATGACAAGGCGAGCCGGCACGCCCCCTGGCTCAAGGGATCCG<br>CCTTTGACAGCATCACCATGGGGGAGCTGGCTACCTACAGCGCGGGCGGCTTGCCGCTGCAATTCCCCGAGGAGGTGGATTCGCTCGAGAAGA<br>TGCAGGCCTACTACCGCCAGTGGACCCCAGCCTACTCGCCGGGTTCCCATCGCCAGTACTCTAACCCCAGCATAGGGCTGTTCGGCCAC CTGG<br>CGGCGAGCAGCATGAAGCAGCCGTTTGCCCAGTTGATGGAGCAGACGCTCCTGCCGGGGCTTGGCCTGCACCACAC
blaSHVII	TTAGCGTTGCCAGTGCTCGATCAGCGCCGCGCCGATCCCGGCGATTTGCTGATTTCGCTCGGCCATGCTCGCCGGCGTATCCCGCAGA<br>TAAATCACCACAATGCGCTCTGCTTTGTTATTCGGGCCAAGCAGGGCGACAATCCCGCGCGCACCCCGCTTGCTAGC-TCCGGTCTTATC<br>GGCGATAAAC-CAGCCCGCCGGCAGCACGGAGCGGATCAACGGTCCGGCGACCCGATCGTCCACCATCCACTGCAGCAGCTGCCGTTG<br>CGAACGGGCGCTCAGACGCTGGCTGGTCAGCAGCTTGCGCAGGGTCGCGGCCATGCTGGCCGGGGTAGTGGTGTCGCGGGCGTCGC<br>CGGGAAGCGCCTCATTCAGTTCCGTTTCCCAGCGGTCAAGGCGGGTGACGTTGTCGCCGATCTGGCGCAAAAAGGCAGTCAATCCTGCG<br>GGGCCGCCGACGGTGGCCAGCAGCAGATTGGCGGCGCTGTTATCGCTCATGGTAATGGCGGCGGCGCAGAGTTCGCCGACCGTCATGC<br>CGTCGGCAAGGTGTTTTTCGCTGACCGGCGAGTAGTCCACCAGATCCTGCTGGCGATAGTGGATCTTTCGCTCCAGCTGTTCGTCACCGG<br>CATCCACCCGCGCCAGCACTGCGCCGCAGAGCACTACTTTAAAGGTGCTCATCATGGGAAAGCGTTCATCGGCGCGCCAGGCGGTCAGC<br>GTGCGGCCGCTGGCCAGATCCATTTCTATCATGCCTACGCGGCCCGACAGCTGGCTTTCGCTTTGTTTAATTTGCTCAAGCGGCTGCGGG<br>CTGGCGTGTACCGCCAGCGGCAGGGTGGCTAACAGGGAGATAATACACAGGCGAATATAACGCAT
blaOXA	ATGAACATTAAAGCACTCTTACTTATAACAAGCGCTATTTTTATTTCAGCCTGCTCACCTTATATAGTGACTGCTAATCCAAATCACAGCGC<br>TTCAAAATCTGATGTAAAAGCAGAGAAAATTAAAAATTTATTTAACGAAGCACACACTACGGGTGTTTTAGTTATCCAACAAGGCCAAACTC<br>AACAAAGCTATGGTAATGATCTTGCTCGTGCTT

blaCTX-M-1	TGCACCGGTGGTATTGCCTTTCATCCATGTCACCAGCTGCGCCCGTTGGCTGTCGCCCAATGCTTTACCCAGCGTCAGATTCCGCAGAGT<br>TTGCGCCATTGCCCG
ermB	ATGAACAAAAATATAAAATATTCTCAAAACTTTTTAACGAGTGAAAAAGTACTCAACCAAATAATAAAACAATTGAATTTAAAAGAAACCGATA
	CCGTTTACGAAATTGGAACAGGTAAAGGGCATTTAACGACGAAACTGGCTAAAATAAGTAAACAGGTAACGTCTATTGAATTAGACAGTCAT<br>CTATTCAACTTATCGTCAGAAAAATTAAAACTGAATACTCGTGTCACTTTAATTCACCAAGATATTCTACAGTTTCAATTCCCTAACAAACAGA<br>GGTATAAAATTGTTGGGAATATTCCTTACCATTTAAGCACACAAATTATTAAAAAAGTGGTTTTTGAAAGCCATGCGTCTGACATCTATCTGA<br>TTGTTGAAGAAGGATTCTACAAGCGTACCTTGGATATTCACC
ermF	TGAAAACGACACAGCTTTGGTTGAACATTTACGAAAATTATTTTCTGATGCCCGAAATGTTCAAGTTGTCGGTTGTGATTTTAGGAATTTTGC<br>AGTTCCG
ermT	AATACAAATCGTTCACTAGCACTATTTTTAATGACAGAAGTTGATATATCCATATTAAGTAAAATCCCTAGAGAATACTTTCATCCAAAACCTA G<br>AGTTAATAGCTCGTTAATTGTATTAAAAAGACACCCTTCAAAAATATCA
ermX	GATGATGACGGCTCAGTGGTCCCCATGGTTCACATTTCACCTGGGTTCTCGGGTACCAAGGTCTGCTTTCCGGCCACAGCCAAACGTTGA CG<br>GGGGGATCTTAGTGATC
erm35	GAATTCAAGTATTCACCTGTAAGAAGTAAATAATGACAAAAAAGAAATTGCCCGTTCGTTTTACGGGTCAGCACTTTACTATTGACAAAGTGCTT<br>ATTAAAGATGCAATAAAAGAATCAAATATAAATCAACACGATACAGTTTTAGATATTGGAGCTGGTAAGGGTTTTCTAACTGTTCATCTCTTAAA<br>A<br>AATGTCGATAAAGTTATTGCCATTGAAAACGATGTTGCATTAAGTCAACATTTGCGCAAAAAATTCATTCACGCTCAAAACGTTCAAGTGGTTAG<br>TTGTGATTATAGAAATTTTGTGGTTCCGAAAGTTCCATTTAAAGTAGTTTCAAATATTCCTTTTGGTATTACATCTGATATTTTTAGTAGTCTG ATG<br>TTTGAAAATGTCGAATATTTTCTATGCGGTTCAATTAT
tet32	TAATCCCATGCCATTACTTCGGACAACGGTAGAGCCGCAAAAGCCGGAGCAAAGGGAAGCCCTGTTAAATGCCCTCACAGAGATTGCTGA<br>TACAGA
tetA	ACTGGCGGCGCTCACCAGCCTGACCTCGATCGTCGGACCCCTCCTCTTCACGGCGATCTATGCGGCTTCTATAACAACGTGGAACGGGT<br>GG
tetL	AGCACTCGTAATGGTTGTAGTTGCGCGCTATATTCCAAAGGAAAATAGGGGTAAAGCATTTGGTCTTATTGGATCGATAGTAGCCATGGGA GAA<br>GGAGTCGGTCCAGCGATTGGTGGAATG
tetO	GCAGGAAAGACAACATTAACGGAAAGTTTATTGTATACCAGTGGTGCAATTGCAGAACTAGGGAGCGTAGATGAAGGCACAACAAGGACA GAT<br>ACAATGAATTTGGAGCGTCAAAGGGGAATCA
tetQ	GTAAAGACTACGCCTCAGAAGTAAGTTCATACACTAAGGGCTTAGGCGTTTTTATGGTCAAGCCATGCGGGTATCAAATAACAAAAGGCGA TTA<br>TTCTGATAATATCCGCATGAACGAAAAAGATAAA
tetS	TATCAAGATATTAAGGACAAACTTTCTGACGACATCATAATTAAGCAGACTGTGAATCTAAATTTGAAACCTTATGTAATAGATTATACTGAA<br>CCA<br>GAACAATGGGAGACAGTAATTGTGG
tetW	CCCTGCGGAAAATGAACATTCCCACCGTTATCTTTATCAACAAGATCGACCAGGTTGGCGTTGATTTGCAGGGCGTGTATCAGTCTGTTCG GG<br>ATAAGCTCTCCGCCGATATTATCATCAAG
tetX	GTTTCCATTGCATAGCTGAAAAAATCCAGGACAGTTTATCTCTGGTTGATGAATATCGGCTTGTATATTGAAAGTACCTGTTTCTTCAACTTC CG<br>TGTCGGTAACAAATTTTCTTACCTTG
tetM	ATGGAGGAAAATCACATGAAAATTATTAATATTGGAGTTTTAGCTCATGTTGATGCGGGAAAAACTACCTTAACAGAAAGCTTATTATATAAC<br>AGT
vanA	TCACCCCTTTAACGCTAATACGATCAAGCGGTCAATCAGTTCGGGAAGTGCAATACCTGCAGCGGCCATCATACGGGGATAACGACTGTA TGA<br>CGTGAAACCGGGCAGAGTATTGACTTCG
vanB	GATGATTTGATTGTCGGCGAAGTGGATCAAATCCGGTTGAGCCACGGTATCTTCCGCATCCATCAGGAAAACGAGCCGGAAAAAGGCTCA<br>GAGAATGC
vanRA	TGCTGAAATATTCGTCGCCCCATATCTCATGAAATAGCAGCTCGGAGCTAACCACATTCCCCTTGTTTTCACAGAGGATTCGCAGTATTGAA AAC<br>TCGGTGGGAGTAAGGGATAACTGCT
vanSA	TATTCTATGTCGCGTCATGCTTTCAAAATTCGCAAAATACTTTGACGAGATAAATACCGGCATTGATGTACTTATTCAGAACGAAGATAAACA AA<br>TTGAGCTTTCTGCGGAAATGGATGTT
sul1	ATGGTGACGGTGTTCGGCATTCTGAATCTCACCGAGGACTCCTTCTTCGATGAGAGCCGGCGGCTAGACCCCGCCGGCGCTGTCACCGC G<br>GCGATCGAAATGCTGCGAGTCGGATCAGACGTCGTGGATGTCGGACCGGCCGCCAGCCATCCGGACGCGAGGCCTGTATCGCCG
sul2	ATGAATAAATCGCTCATCATTTTCGGCATCGTCAACATAACCTCGGACAGTTTCTCCGATGGAGGCCGGTATCTGGCGCCAGACGCAGCC<br>ATTGCGCAGGCGCGTAAGCTGATGGCCGAGGGGGCAGATGTGATCGACCTCGGTCCGGCATCCAGCAATCCCGACGCCGCGCCTGTTT<br>CGTCCGACACAGAAATCGCGCGTATCGCGCCGGTGCTGGACGCGCTCAAGGCAGATGGCATTCCCGTCTCGCTCGACAGTTATCAACCC<br>GCGACGCAAGCCTATGCCTTGTCGCGTGGTGTGGCCTATCTCAATGATATTCGCGGTTTTCCAGACGCTGCGTTCTATCCGCAATTGGCG AAA
qnrB	TTAACCCATGACAGCGATACCAAGACGTTCCAGGAGCAACGATGCCTGGTAGCTGTCCAGTTTGACGCCTTGCAAATCAACCCCGC
mcr-1	ATGGCACGGTCTATGATACGACCATGCTCCAAAATGCCCTACAGACCGACCAAGCCGAGACCAAGGATCTATTAAACGCAGCGTTTATCA<br>TGCGTATCATTGGTTTGGGTGTGCTACCAAGTTTGCTTGTGGCTTTTGTTAAGGTGGATTATCCG
is26	TTACATTTCAAAAACTCTGCTTACCAGGCGCATTTCGCCCAGGGGATCACCATAATAAAATGCTGAGGCCTGGCCTTTGCGTAGTG<br>CACGCATCACCTCAATACCTTTGATGGTGGCGTAAGCCGTCTTCATGGATTTAAATCCCAGCGTGGCGCCGATTATCCGTTTCAGT<br>TTGCCATGATCGCATTCAATCACGTTGTTC
tnpA	TTTATCGTGCAATTGATGCGGACGGCTTAACCTTAGATATCTGGTTACGAAAGAAATGGGATACGCAAGCAGCCTATGCTTTCTTAA<br>AACGACTCCATAAACAGTTTGGTGAGCCGAAAGCA
int1-A	ATGAAAACCGCCACTGCGCCGTTACCACCGCTGCGTTCGGTCAAGGTTCTGGACCAGTTGCGTGAGCGCATACGCTACTTGCATT<br>ACAGCTTACGAACCGAACAGGCTTATGTCCACTGGGTTCGTGCCTTCATCCGTTTCCACGGTGTGCGTCACCCGGCAACCTTGGGCAGCA<br>G

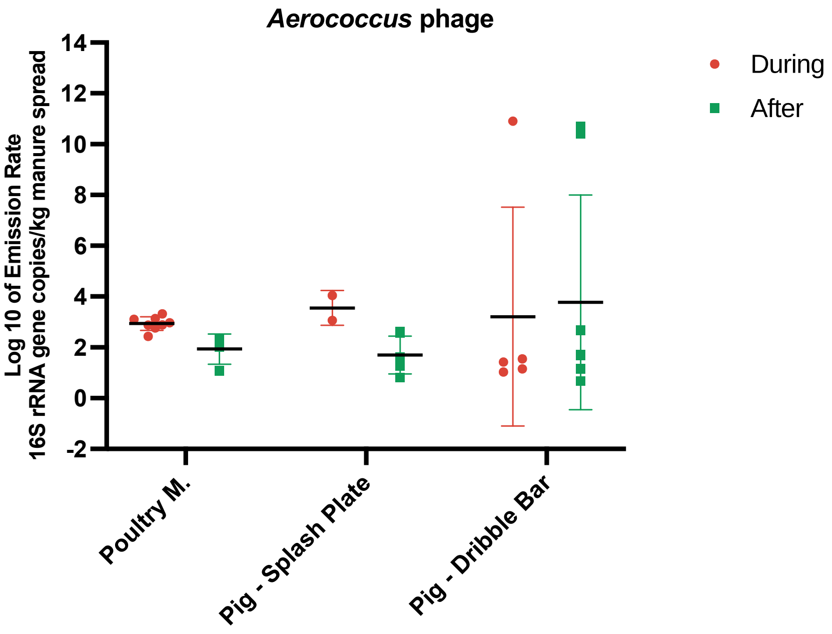

Figure S3. Emission rate of the phage *vB_AviM_AVP* of *Aerococcus* viridans for tested manure types; No difference was observed among the paired groups regarding emission rates of the phage.

References

20. Desbiens, V.; Brassard, P.; Baghdadi, M.; Létourneau, V.; Turgeon, N.; Duchaine, C.; Trivino Arevalo, A.; Godbout, S. Comparison of Air Contaminants Emissions from Two Pig Slurry Spreading Methods in a Controlled Environment—Technical Library of the CSBE-SCGAB. Available online: https://library.csbe-scgab.ca/all-publications/5968:comparison-of-air-contaminants-emissions-from-two-pig-slurry-spreading-methods-in-a-controlled-environment (accessed on 23 January 2023).
22. Stedtfeld, R.D.; Guo, X.; Stedtfeld, T.M.; Sheng, H.; Williams, M.R.; Hauschild, K.; Gunturu, S.; Tift, L.; Wang, F.; Howe, A.; et al. Primer Set 2.0 for Highly Parallel QPCR Array Targeting Antibiotic Resistance Genes and Mobile Genetic Elements. *FEMS Microbiol. Ecol.* **2018**, *94*, fiy130. https://doi.org/10.1093/FEMSEC/FIY130.
44. Oliver, J.P.; Gooch, C.A.; Lansing, S.; Schueler, J.; Hurst, J.J.; Sassoubre, L.; Crossette, E.M.; Aga, D.S. Invited Review: Fate of Antibiotic Residues, Antibiotic-Resistant Bacteria, and Antibiotic Resistance Genes in US Dairy Manure Management Systems. *J. Dairy Sci.* **2020**, *103*, 1051–1071. https://doi.org/10.3168/JDS.2019-16778.
45. Bach, H.J.; Tomanova, J.; Schloter, M.; Munch, J.C. Enumeration of Total Bacteria and Bacteria with Genes for Proteolytic Activity in Pure Cultures and in Environmental Samples by Quantitative PCR Mediated Amplification. *J. Microbiol. Methods* **2002**, *49*, 235–245. https://doi.org/10.1016/S0167-7012(01)00370-0.
46. Malinen, E.; Kassinen, A.; Rinttilä, T.; Palva, A. Comparison of Real-Time PCR with SYBR Green I or 5′-Nuclease Assays and Dot-Blot Hybridization with RDNA-Targeted Oligonucleotide Probes in Quantification of Selected Faecal Bacteria. *Microbiology* **2003**, *149*, 269–277. https://doi.org/10.1099/MIC.0.25975-0.
47. Baker, G.C.; Smith, J.J.; Cowan, D.A. Review and Re-Analysis of Domain-Specific 16S Primers. *J. Microbiol. Methods* **2003**, *55*, 541–555. https://doi.org/10.1016/j.mimet.2003.08.009.
48. Robb, F.T.; DasSarma, S.; Fleischmann, E.M. *Archaea : A Laboratory Manual*; Cold Spring Harbor Laboratory: New York, NY, USA, 1995; pp. 217.
